# Supplementary material for: Transcriptome profiling reveals stress-responsive gene networks in cattle muscles
Source: PeerJ. 2022 Apr 6;10:e13150. doi: 10.7717/peerj.13150 (PMC8994496; doi:10.7717/peerj.13150)
Supplement: Supplemental Information 3 — The sequence of the primers was designed using the Primer 3 software. [file peerj-10-13150-s003.docx]

**Supplemental Table S1:**

**Sequence of the primers used in qPCR experiments.**

The sequence of the primers was designed using the Primer 3 software.

| **Gene Name** | **Forward primer** | **Reverse primer** | **Tm** |
| --- | --- | --- | --- |
| **ATF3** | gAg CTA AgC AgC CgT ggT AT | ggC TTC Agg gTT TCg gAT gT | 60 |
| **FOS** | gCA AAA CgC ATg gAg TgT gT | AAA AgA gAC gCA gAC CCA gg | 60 |
| **CEBPD** | gTT gCC TTT CCC CCA TgA gA | ggA ggT AgC AgC CAA gAC AA | 60 |
| **SMAD7** | CTT AgC CgA CTC TgC gAA CT | gCA CAg CAT CTg gAC AgT CT | 60 |
| **TOP2B** | CCg ATg ATg ATg ACg ACA AT | TgC TAT ggg AgA TgC TTT gA | 60 |
| **UXT** | TgT ggC CCT Tgg ATA Tgg TT | ggT TgT CgC TgA gCT CTg Tg | 60 |
| **MRPL39** | TTg gTC AgA gCC CCA gAA gT | Agg TTC TCT TTT gTT ggC ATC C | 62 |
| **CLN3** | TTC TgA CTC CTT ggg ACA CA | CAA CCT gCC CAC CTA TCA gT | 58 |
