# Supplementary material for: Transcriptome profiling reveals stress-responsive gene networks in cattle muscles
Source: PeerJ. 2022 Apr 6;10:e13150. doi: 10.7717/peerj.13150 (PMC8994496; doi:10.7717/peerj.13150)
Supplement: Supplemental Information 4 — Differential expression was computed on the GSE119912 using the LIMMA method with a Benjamini–Hochberg (BH) multiple testing correction. Genes for which 80% were differential probes at the adjusted p-value BH<10% were retained. [file peerj-10-13150-s004.docx]

**Supplemental Table S2:**

**Differential genes in response to stress in the *Longisssimus thoracis* (LT) muscle and *Semitendinosus* (ST) muscle in stressed cows compared to controls.**

Differential expression was computed on the GSE119912 using the LIMMA method with a Benjamini-Hochberg (BH) multiple testing correction. Genes for which 80% were differential probes at the adjusted p-value BH<10% were retained.

| **Muscle** | **Symbol** | **Description** | **Gene Name** | **Min.Ratio. estimate** | **Min.p-value BH** | **Max.Ratio.estimate** | **Max p-value BH** | **Mean ratio (stressed vs controls)** |
| --- | --- | --- | --- | --- | --- | --- | --- | --- |
| **LT** | MYOG | Myogenin (TF) | **MYOG** | 0.48 | **<0.005** | 0.49 | **<0.005** | 0.48 |
|  | CXCR6 | chemokine receptor 6 | **CXCR6** | 0.49 | **<0.005** | 0.49 | **<0.005** | 0.49 |
|  | LOC518746 | Bos taurus similar to Homo sapiens delta-like 4 | **DLL4** | 0.51 | **<0.005** | 0.51 | **<0.005** | 0.51 |
|  | LEAP2 | liver expressed antimicrobial peptide 2 | **LEAP2** | 0.61 | **<0.005** | 0.61 | **<0.005** | 0.61 |
|  | MYOD1 | myogenic differentiation 1 (TF) | **MYOD1** | 0.60 | **0.02** | 0.66 | 0.12 | 0.63 |
|  | SLC2A3 | solute carrier family 2 member 3 | **SLC2A3** | 0.62 | **0.02** | 0.67 | 0.09 | 0.64 |
|  | AU231904 | Bos taurus similar to Homo sapiens solute carrier family. member 3 | **SLC2A3** | 0.65 | **0.05** | 0.65 | **0.05** | 0.65 |
|  | HES1 | hairy and enhancer of split 1 (TF) | **HES1** | 0.65 | 0.07 | 0.65 | 0.07 | 0.65 |
|  | BC120165 | Bos taurus HSPB (heat shock 27kDa) associated protein 1 mRNA | **HSPB1** | 0.59 | **<0.005** | 0.73 | **0.04** | 0.67 |
|  | LOC506314 | Bos taurus similar to Homo sapiens interleukin 16 transcript variant 1 | **IL16** | 0.68 | **0.03** | 0.68 | **0.03** | 0.68 |
|  | ARL6IP2 | ADP-ribosylation factor-like 6 interacting protein 2 | **ARL6IP2** | 0.68 | **0.01** | 0.68 | **0.01** | 0.68 |
|  | XYLT2 | xylosyltransferase II | **XYLT2** | 0.69 | **0.02** | 0.69 | **0.02** | 0.69 |
|  | PIGM | phosphatidylinositol glycan anchor biosynthesis class M | **PIGM** | 0.69 | **0.01** | 0.69 | **0.01** | 0.69 |
|  | LOC511560 | Bos taurus similar to Homo sapiens IMP3 U3 small nucleolar ribonucleoprotein homolog | **IMP3** | 0.71 | **0.03** | 0.71 | **0.03** | 0.71 |
|  | LOC523999 | Bos taurus similar to Homo sapiens phosphatidylinositol transfer protein membrane-associated 2 | **PITPNM2** | 0.73 | 0.06 | 0.73 | 0.06 | 0.73 |
|  | RAB3IL1 | RAB3A interacting protein (rabin3)-like 1 | **RAB3IL1** | 0.72 | **0.01** | 0.76 | 0.08 | 0.74 |
|  | NME6 | non-metastatic cells 6 protein expressed in (nucleoside-diphosphate kinase) | **NME6** | 0.76 | 0.07 | 0.76 | 0.07 | 0.76 |
|  | LOC614335 | Bos taurus similar to Homo sapiens RAB3A interacting protein-like 1 | **RAB3IL1** | 0.76 | 0.07 | 0.76 | 0.07 | 0.76 |
|  | BE663779 | Bos taurus similar to Homo sapiens nucleolar protein family 6 (RNA-associated) transcript variant alpha | **NOL6** | 0.76 | 0.08 | 0.76 | 0.08 | 0.76 |
|  | CDIPT | CDP-diacylglycerol--inositol 3-phosphatidyltransferase | **CDIPT** | 0.76 | **<0.005** | 0.76 | **<0.005** | 0.76 |
|  | TREM1 | triggering receptor expressed on myeloid cells 1 | **TREM1** | 0.77 | **0.02** | 0.77 | **0.02** | 0.77 |
|  | LOC615759 | Bos taurus similar to Superfast myosin regulatory light chain 2 | **MYLC2** | 0.77 | **0.05** | 0.77 | **0.05** | 0.77 |
|  | LOC515906 | Bos taurus similar to Homo sapiens integrin alpha E | **ITGAE** | 0.78 | **0.03** | 0.78 | **0.03** | 0.78 |
|  | DFFB | DNA fragmentation factor 40kDa beta polypeptide | **DFFB** | 0.78 | 0.08 | 0.78 | 0.08 | 0.78 |
|  | ACOT11 | acyl-CoA thioesterase 11 | **ACOT11** | 1.28 | 0.10 | 1.28 | 0.10 | 1.28 |
|  | ATP1B1 | ATPase Na /K transporting beta 1 polypeptide | **ATP1B1** | 1.35 | 0.09 | 1.35 | 0.09 | 1.35 |
|  | PGF | placental growth factor vascular endothelial growth factor-related protein | **PGF** | 1.36 | **0.05** | 1.36 | **0.05** | 1.36 |
|  | CEBPB | CCAAT/enhancer binding protein beta (TF) | **CEBPB** | 0.85 | **<0.005** | 1.64 | 0.45 | 1.39 |
|  | LOC534206 | Bos taurus similar to Homo sapiens tubulin beta 6 | **TUBB6** | 1.41 | 0.06 | 1.41 | 0.06 | 1.41 |
|  | MUSK | muscle skeletal receptor tyrosine kinase | **MUSK** | 1.42 | **0.04** | 1.42 | **0.04** | 1.42 |
|  | LOC615966 | Bos taurus similar to Homo sapiens tyrosine 3-monooxygenase/tryptophan 5-monooxygenase activation protein zeta polypeptide transcript variant 1 | **YWHAZ** | 1.43 | **0.03** | 1.43 | **0.03** | 1.43 |
|  | IDS | iduronate 2-sulfatase (Hunter syndrome) | **IDS** | 1.43 | **0.02** | 1.43 | **0.02** | 1.43 |
|  | PLD1 | phospholipase D1 phosphatidylcholine-specific | **PLD1** | 1.44 | **0.03** | 1.44 | **0.03** | 1.44 |
|  | HEYL | hairy/enhancer-of-split related with YRPW motif-like (TF) | **HEYL** | 1.47 | **<0.005** | 1.47 | **<0.005** | 1.47 |
|  | AB257561.1 | Bos taurus MRF4 mRNA for myogenic regulatory factor 4 (TF) | **MYF6** | 1.44 | 0.07 | 1.51 | 0.14 | 1.48 |
|  | LOC541274 | Bos taurus similar to Homo sapiens DnaJ (Hsp40) homolog ubfamily B member 4 | **DNAJB4** | 1.50 | **0.02** | 1.50 | **0.02** | 1.50 |
|  | SLC25A25 | solute carrier family 25 member 25 | **SLC25A25** | 1.47 | **0.03** | 1.63 | **0.04** | 1.54 |
|  | MED23 | mediator complex subunit 23. transcriptional co-factor | **MED23** | 1.55 | **0.03** | 1.55 | **0.03** | 1.55 |
|  | DNAJB4 | DnaJ (Hsp40) homolog subfamily B member 4 | **DNAJB4** | 1.53 | **0.01** | 1.58 | **0.03** | 1.55 |
|  | LCAT | lecithin-cholesterol acyltransferase | **LCAT** | 1.48 | **<0.005** | 1.66 | **0.03** | 1.57 |
|  | PMP22 | peripheral myelin protein 22 | **PMP22** | 1.51 | **<0.005** | 1.67 | 0.07 | 1.60 |
|  | SMAD7 | SMAD family member 7. transcription co-factor | **SMAD7** | 1.53 | **<0.005** | 1.78 | 0.11 | 1.68 |
|  | THBS1 | thrombospondin 1 | **THBS1** | 1.65 | **0.03** | 1.74 | 0.07 | 1.70 |
|  | ADRB2 | adrenergic beta-2- receptor surface | **ADRB2** | 1.59 | **<0.005** | 1.75 | 0.08 | 1.70 |
|  | SORBS1 | sorbin and SH3 domain containing 1 | **SORBS1** | 1.66 | **<0.005** | 1.90 | **<0.005** | 1.80 |
|  | LOC286844 | pyruvate dehydrogenase phosphatase regulatory subunit precursor | **PDPR** | 1.85 | 0.06 | 1.85 | 0.06 | 1.85 |
|  | ABRA | actin-binding Rho activating protein | **ABRA** | 1.86 | **<0.005** | 1.86 | **<0.005** | 1.86 |
|  | GPAM | glycerol-3-phosphate acyltransferase itochondrial | **GPAM** | 1.87 | **<0.005** | 1.87 | **<0.005** | 1.87 |
|  | LOC538437 | Bos taurus similar to Homo sapiens GTP binding protein overexpressed in skeletal muscle transcript variant 1 | **GEM** | 1.88 | **<0.005** | 1.88 | **<0.005** | 1.88 |
|  | LOC504317 | Bos taurus similar to Homo sapiens low density lipoprotein receptor-related protein 4 | **LRP4** | 1.91 | **0.01** | 1.91 | **0.01** | 1.91 |
|  | GLUL | glutamate-ammonia ligase | **GLUL** | 1.76 | **<0.005** | 2.13 | **<0.005** | 1.93 |
|  | GADD45A | growth arrest and DNA-damage-inducible alpha | **GADD45A** | 2.06 | **<0.005** | 2.06 | **<0.005** | 2.06 |
|  | ETS2 | v-ets erythroblastosis virus E26 oncogene homolog 2 (TF) | **ETS2** | 2.02 | **<0.005** | 2.17 | **<0.005** | 2.10 |
|  | SERPINE1 | serpin peptidase inhibitor clade E member 1 | **SERPINE1** | 1.68 | **0.01** | 2.28 | 0.21 | 2.13 |
|  | LOC531384 | Bos taurus similar to Homo sapiens hairy and enhancer of split 6 (TF) | **HES6** | 2.24 | **<0.005** | 2.24 | **<0.005** | 2.24 |
|  | CB465209 | Bos taurus similar to Homo sapiens sparc/osteonectin. cwcv and kazal-like domains proteoglycan 2 | **SPOCK2** | 2.31 | **0.04** | 2.31 | **0.04** | 2.31 |
|  | SDC4 | syndecan 4 | **SDC4** | 2.23 | **<0.005** | 2.36 | **<0.005** | 2.32 |
|  | SLC16A6 | solute carrier family 16 member 6 | **SLC16A6** | 2.25 | **<0.005** | 2.59 | **<0.005** | 2.39 |
|  | FOS | v-fos FBJ murine osteosarcoma viral oncogene homolog (TF) | **FOS** | 2.46 | **0.02** | 2.60 | **0.03** | 2.53 |
|  | IFRD1 | interferon-related developmental regulator 1 | **IFRD1** | 2.38 | **<0.005** | 2.79 | **<0.005** | 2.60 |
|  | PFKFB3 | 6-phosphofructo-2-kinase/fructose-2 6-biphosphatase 3 | **PFKFB3** | 0.79 | **<0.005** | 4.52 | 0.54 | 2.60 |
|  | LOC515266 | Bos taurus similar to Homo sapiens activating transcription factor 3 transcript variant 1 (TF) | **ATF3** | 2.61 | **<0.005** | 2.61 | **<0.005** | 2.61 |
|  | S77845 | Bos taurus similar to Homo sapiens 6-phosphofructo-2-kinase/fructose-2.6-biphosphatase 3 | **PFKFB3** | 2.89 | **<0.005** | 2.89 | **<0.005** | 2.89 |
|  | CEBPD | CCAAT/enhancer binding protein delta (TF) | **CEBPD** | 3.17 | **<0.005** | 4.09 | **<0.005** | 3.61 |
|  | RGS2 | regulator of G-protein signaling 2 24kDa | **RGS2** | 3.53 | **<0.005** | 4.09 | **<0.005** | 3.83 |
|  | PDK4 | pyruvate dehydrogenase kinase isozyme 4 | **PDK4** | 7.84 | **<0.005** | 10.62 | **0.00** | 8.77 |
|  | CB463123 | Bos taurus similar to Homo sapiens pyruvate dehydrogenase kinase isozyme 4 | **PDK4** | 9.91 | **<0.005** | 9.91 | **0.00** | 9.91 |
| **ST** | MYOG | myogenin (myogenic factor 4) (TF) | **MYOG** | 0.56 | **<0.005** | 0.59 | **<0.005** | 0.57 |
|  | LOC540257 | similar to Serine/threonine-protein phosphatase 2B catalytic subunit beta isoform (Calmodulin-dependent calcineurin A subunit beta isoform) | **PPP2B** | 0.60 | **<0.005** | 0.94 | 1.00 | 0.69 |
|  | ARL6IP2 | ADP-ribosylation factor-like 6 interacting protein 2 | **ARL6IP2** | 0.72 | **0.01** | 0.72 | **<0.005** | 0.72 |
|  | TUBB6 | tubulin beta 6 | **TUBB6** | 0.96 | **0.01** | 1.56 | 1.00 | 1.38 |
|  | HYAL2 | hyaluronoglucosaminidase 2 | **HYAL2** | 1.41 | **0.02** | 1.41 | **0.02** | 1.41 |
|  | HEYL | hairy/enhancer-of-split related with YRPW motif-like (TF) | **HEYL** | 1.48 | 0.06 | 1.48 | 0.06 | 1.48 |
|  | SLC2A8 | solute carrier family 2 member 8 | **SLC2A8** | 1.50 | 0.06 | 1.50 | 0.06 | 1.50 |
|  | PGF | placental growth factor vascular endothelial growth factor-related protein | **PGF** | 1.51 | **0.02** | 1.51 | **0.02** | 1.51 |
|  | LOC534206 | Bos taurus similar to Homo sapiens tubulin beta 6 | **TUBB6** | 1.54 | **0.02** | 1.54 | **0.02** | 1.54 |
|  | ABRA | actin-binding Rho activating protein | **ABRA** | 1.54 | 0.06 | 1.54 | 0.06 | 1.54 |
|  | LCAT | lecithin-cholesterol acyltransferase | **LCAT** | 1.47 | **0.01** | 1.59 | **0.03** | 1.55 |
|  | LOC504317 | Bos taurus similar to Homo sapiens low density lipoprotein receptor-related protein 4 | **LRP4** | 1.59 | 0.06 | 1.59 | 0.06 | 1.59 |
|  | SMAD7 | SMAD family member 7. transcription co-factor | **SMAD7** | 1.60 | **0.01** | 1.67 | **0.02** | 1.63 |
|  | LOC538437 | Bos taurus similar to Homo sapiens GTP binding protein overexpressed in skeletal muscle transcript variant 1 | **GEM** | 1.65 | **0.01** | 1.65 | **<0.005** | 1.65 |
|  | SORBS1 | sorbin and SH3 domain containing 1 | **SORBS1** | 1.55 | **<0.005** | 1.80 | **0.03** | 1.70 |
|  | MYLK4 | myosin light chain kinase family member 4 | **MYLK4** | 1.84 | **<0.005** | 1.84 | **<0.005** | 1.84 |
|  | PFKFB3 | 6-phosphofructo-2-kinase/fructose-2 6-biphosphatase 3 | **PFKFB3** | 1.03 | **<0.005** | 2.53 | 1.00 | 1.92 |
|  | SDC4 | syndecan 4 | **SDC4** | 1.92 | **0.04** | 1.97 | **0.05** | 1.94 |
|  | ZNF750 | zinc finger protein 750 | **ZNF750** | 1.99 | **0.05** | 1.99 | **0.05** | 1.99 |
|  | CYP1A1 | cytochrome P450 subfamily I polypeptide 1 | **CYP1A1** | 1.90 | **<0.005** | 2.13 | **0.02** | 2.00 |
|  | S77845 | Bos taurus similar to Homo sapiens 6-phosphofructo-2-kinase/fructose-2.6-biphosphatase 3 | **PFKFB3** | 2.00 | **<0.005** | 2.00 | **<0.005** | 2.00 |
|  | PMP22 | peripheral myelin protein 22 | **PMP22** | 1.99 | **<0.005** | 2.06 | **<0.005** | 2.02 |
|  | ADAMTS9 | ADAM metallopeptidase with thrombospondin type 1 motif 9 | **ADAMTS9** | 2.26 | **0.01** | 2.26 | **0.01** | 2.26 |
|  | GADD45A | growth arrest and DNA-damage-inducible alpha | **GADD45A** | 2.28 | **<0.005** | 2.28 | **<0.005** | 2.28 |
|  | ETS2 | v-ets erythroblastosis virus E26 oncogene homolog 2 (TF) | **ETS2** | 2.31 | **<0.005** | 2.39 | **<0.005** | 2.35 |
|  | LOC531384 | Bos taurus similar to Homo sapiens hairy and enhancer of split 6 (TF) | **HES6** | 2.45 | **<0.005** | 2.45 | **<0.005** | 2.45 |
|  | IFRD1 | interferon-related developmental regulator 1 | **IFRD1** | 2.41 | **<0.005** | 2.69 | **<0.005** | 2.50 |
|  | SLC16A6 | solute carrier family 16 member 6 | **SLC16A6** | 2.66 | **<0.005** | 3.16 | **<0.005** | 2.99 |
|  | CEBPD | CCAAT/enhancer binding protein delta (TF) | **CEBPD** | 2.71 | **<0.005** | 3.35 | **<0.005** | 3.00 |
|  | FOS | v-fos FBJ murine osteosarcoma viral oncogene homolog (TF) | **FOS** | 3.05 | **<0.005** | 3.15 | **0.01** | 3.09 |
|  | SPOCK2 | sparc/osteonectin cwcv and kazal-like domains proteoglycan 2 | **SPOCK2** | 2.85 | **<0.005** | 3.59 | **<0.005** | 3.19 |
|  | CB465209 | Bos taurus similar to Homo sapiens sparc/osteonectin. cwcv and kazal-like domains proteoglycan 2 | **SPOCK2** | 3.38 | **<0.005** | 3.38 | **<0.005** | 3.38 |
|  | LOC515266 | Bos taurus similar to Homo sapiens activating transcription factor 3 transcript variant 1 (TF) | **ATF3** | 3.84 | **<0.005** | 3.84 | **<0.005** | 3.84 |
|  | RGS2 | regulator of G-protein signaling 2 4kDa | **RGS2** | 4.64 | **<0.005** | 5.15 | **<0.005** | 4.94 |
|  | PDK4 | pyruvate dehydrogenase kinase isozyme 4 | **PDK4** | 8.76 | **<0.005** | 10.66 | **<0.005** | 9.46 |
|  | CB463123 | Bos taurus similar to Homo sapiens pyruvate dehydrogenase kinase isozyme 4 | **PDK4** | 10.68 | **<0.005** | 10.68 | **<0.005** | 10.68 |

TF: Transcription factor

Min.Ratio. estimate: minimum (stressed/not stressed) ratio estimate among differentially expressed probes (BH 10%) for the concerned gene

Min.p-value BH: minimum BH p-value among differentially expressed probes (BH 10%) for the concerned gene

Max.Ratio. estimate: maximum (stressed/not stressed) ratio estimate among differentially expressed probes (BH 10%) for the concerned gene

Max.p-value BH: maximum BH p-value among differentially expressed probes (BH 10%) for the concerned gene

Mean ratio (stressed vs controls): mean (stressed/not stressed) ratio estimate among differentially expressed probes (BH 10%) for the concerned gene
