# Supplementary material for: Transcriptome profiling reveals stress-responsive gene networks in cattle muscles
Source: PeerJ. 2022 Apr 6;10:e13150. doi: 10.7717/peerj.13150 (PMC8994496; doi:10.7717/peerj.13150)
Supplement: Supplemental Information 5 — Promoters of the differentially expressed genes (DEG) were examined for transcriptional cis- modules using the Genomatix software suite. Detection of over-represented modules in the DEG was made by comparison to the total genes of the microarray. [file peerj-10-13150-s005.docx]

**Supplemental Table S3**:

**Over-represented transcriptional cis-modules in the dataset of stress responsive genes in the LT and in the ST muscles**

Promoters of the differentially expressed genes (DEG) were examined for transcriptional cis- modules using the Genomatix software suite. Detection of over-represented modules in the DEG was made by comparison to the total genes of the microarray.

|  | **LT muscle** | | **ST muscle** | |
| --- | --- | --- | --- | --- |
|  | Dataset (DEG) | All genes (microarray) | Dataset (DEG) | All genes (microarray) |
| **Gene ID inputs** | 62 | 4 188 | 32 | 4 117 |
| **Gene ID found in Genomatix Suite** | 52 | 2 594 | 28 | 2 538 |
| **Promoters** | 168 | 7 805 | 84 | 9 124 |
| **Selected promoters** | 111 | 7 805 | 57 | 9 124 |
| **Module locations** | 1 378 | 86 021 | 675 | 84 563 |
| **Different modules** | 288 | 606 | 201 | 605 |
| **Maximum occurrence of a module** | 60 | 3 555 | 32 | 3 506 |
| **Over represented modules** | **24** | | **25** | |
| **Common modules** | **9** | | | |
